# Case of Hanging, Treated Successfully by the Affusion of Cold Water

**Published:** 1846-06

**Authors:** E. Noyce


*Case of Hanging, treated successfully by the affusion of cold water.* —E. Noyce.—At about 3 a. m., a few weeks since, I was sent for to see a man who had suspended himself by the neck by means of a pair of braces. On arriving, I found he had been 'cut down' by his father about four or five minutes before, and from what I could gather, he had been hanging about two or three minutes, so that from the time of suspension to the time of my arrival, at least six minutes must have elapsed, during which time he had ceased to breathe. His features were pallid, and the conjunctiva injected with blood; the heart's action continued, although but feeble, the pulse being about 80, and very weak. I endeavored to perform artificial respiration; but the only pair of bellows I could obtain were so much out of order that this was impossible; I therefore threw them aside, and tried the effect of cold water, well knowing the powerful reflex action on the muscles of inspiration which follows its sudden application to the surface. On the first affusion I perceived a gurgling in the throat, and renewed the operation; a slight inspiration directly succeeded. The affusion was frequently repeated, and was followed each time by an inspiration, each more perfect than the last, until the lungs were fully inflated; respiration then continued regularly, accompanied by frequent yawning and sighing.

I bled him to sixteen ounces, which restored him to consciousness, and he has had no bad symptoms since, excepting a severe pain at the back of the neck, which shortly passed off, and he remains quite well.—*Med. Gaz.*

